# Energy Dissipation and Dynamic Fragmentation of Alkali-Activated Rubber Mortar under Multi-Factor Coupling Effect

**DOI:** 10.3390/ma15217718

**Published:** 2022-11-02

**Authors:** Yuhang Shi, Qinyong Ma, Zifang Xu, Dongdong Ma, Xuan Yang, Yuqi Gu

**Affiliations:** 1 School of Civil Engineering and Architecture, Anhui University of Science and Technology, Huainan 232001, China; 2 School of Material Science and Engineering, Anhui University of Science and Technology, Huainan 232001, China

**Keywords:** recycled rubber aggregate, separating Hopkinson pressure bar, dynamic energy absorption, crushing morphology characteristics, orthogonal analysis

## Abstract

Recycled rubber aggregate (RRA) made from ground tire rubber has been promoted for its light weight and shock resistance. The high alkalinity of alkali-activated slag mortar has a modification effect on the surface of RRA. This paper studies the performance of alkali-activated slag mortar using RRA as aggregate (RASM), which has significance for applications in low-carbon building materials. The orthogonal test analysis method was used to analyze the significance and correlation of the main variables of the test. The dynamic energy absorption capacity and crushing state of RASM under the synergistic effect of various factors were studied using the separating Hopkinson pressure bar (SHPB) test system. The energy absorption characteristics and failure modes of RASM were analyzed by SEM and microscopic pore characterization. The results show that the increase of the alkali equivalent of the mix ratio will increase the peak value of the absorption energy of the specimen. When the size of the RRA is between 0.48 mm~0.3 mm, the dynamic energy absorption of the specimen will reach its peak value. Although the increase in the total volume of RRA will reduce the energy absorption capacity of RASM specimens, its crack resistance is enhanced.

## 1. Introduction

RRA is a rubber powder that can be applied in construction by crushing and grinding tires into sufficient fineness. It is mainly used as an environmentally friendly fine aggregate in cement-based materials. Cement-based materials containing RRA have good performance in fatigue resistance, crack resistance, impact resistance, etc. Therefore, RRA as a building material has broad application prospects in the fields of earthquake resistance, road resistance and explosion resistance [1], and can alleviate the environmental problems caused by the excessive exploitation of river sand caused by the increasing shortage of natural sand. RRA replacing natural sand can increase the fluidity of mortar and reduce the water demand of mortar [2]. With the increase of the volume proportion of RRA in cement-based materials, the mechanical properties of the materials will be reduced, but the toughness and durability of cement-based materials can be improved [3,4,5].

The hydrophilicity of RRA is poor, not only because rubber is a hydrophobic material but also because of its production process. To improve production quality, manufacturers often add zinc stearate and other release agents, further reducing the hydrophilicity of RRA [6,7]. One of the critical defects of RRA, which is used as an aggregate in mortar, is that the strength of the interface transition zone (ITZ) between rubber and cement is low [2]. Moreover, RRA often adsorbs many bubbles, increasing mortar’s porosity. To solve the above problems, modifying the RRA surface is valuable [8,9]. In many modification methods, the use of alkali to remove zinc stearate on the surface of the rubber is a low-difficulty and effective method. In traditional cement base materials, such as ordinary Portland cement (OPC), the pore solution during hydration is less alkaline and it cannot effectively remove zinc stearate from the RRA surface [10,11,12]. Therefore, the selection of cementified materials with strong alkalinity can not only modify the surface of RRA but also eliminate the step of aggregate pretreatment, which can save costs and improve production efficiency.

Alkali-activated slag, as a highly alkaline cementitious material, is widely used in rescue and relief projects and has the fast hard early strength properties of cementitious materials, such as temporary support and bolt grouting [13,14]. At the same time, the granulated blast furnace slag powder, as a substitute for ordinary Portland cement, can be used as an auxiliary cementitious material to partly replace OPC, to reduce the cost of the structure, reduce carbon emissions, and avoid the overuse of high-energy Portland cement [15,16].

In cement-based materials, the contribution of RRA to the resistance of the long-term static load is insufficient, and its main advantage lies in its resistance to dynamic load and cyclic load [3,17,18]. Therefore, in the literature, the research conclusions on the static load of RRA are relatively negative, i.e., RRA will reduce the static compressive strength of cement-based materials. However, some improvements have been shown in studying the dynamic mechanical properties of RRA cement-based materials [19,20]. Therefore, it is of positive significance to study the effect of RRA on the dynamic mechanical properties of alkali-excited slag mortar (ASM).

In the study of the dynamic mechanics of cement-based materials, SHPB (split Hopkinson pressure bar test system) is a practical test method [21,22]. The dynamic loading of ASM specimens using SHPB systems can not only analyze the influence of RRA on mechanical properties and energy dissipation of ASM, but, at the same time, the final failure mode and fractal dimensions of the specimen can be further analyzed [23,24]. Feng, W. et al. [25] investigated the dynamic flexural properties of rubber concrete. Under a dynamic flexural load, rubber particles can reduce the brittleness of concrete without excessive mixing. Gao, Y. et al. [26] carried out an experimental investigation on static and dynamic mechanical properties of high early strength alkali-activated slag concrete, and their results showed that it has high deformability and good impact toughness. Xin, L. et al. [27] showed that the alkali-activator types have a big impact on the dynamic compressive deformation behavior of geopolymer concrete, and, compared to the alkali-activator prepared with NaOH and Na_2_CO_3_, the alkali-activator prepared with NaOH and sodium silicate can be beneficial in the deformation properties.

In previous studies, multi-dimensional experiments and quantitative analysis have been carried out on the impact resistance of rubber concrete made of ordinary Portland cement. However, the energy absorption characteristics and failure modes of alkali-activated slag materials and RRA under dynamic impact load have not been analyzed in detail. At the same time, it is also valuable to study the size and dosage of RRA, as well as the influence of an alkaline environment on RRA on the peak energy absorption of specimens.

In this study, the dynamic loading test, micropore test, microscopic morphology observation, and fracture morphology analysis of ASM using RRA (RASM) were carried out by orthogonal experiments. The main test variables affecting the energy dissipation performance of RASM were systematically evaluated, the optimal mix ratio was determined, and the correlation and significance between each experimental factor and the trend of peak energy consumption data were determined. This article provides a reference for the mixture design of RASM specimens in seismic engineering and road engineering.

## 2. Materials and Methods

### 2.1. Mix Design and Mortar Preparation

The RRA used in the test was prepared from scrap rubber tires after crushing, screening and washing. The granulated blast furnace slag powder was S95 grade. NaOH was solid powder form and the sand was natural river sand. The solid NaOH was completely dissolved using city tap water and the mixed activator was cooled to 23 ℃. XRF detection was carried out on slag powder and RRA, and the results are shown in Table 1 and Table 2.

When preparing mortar, add slag powder, sand and RRA into the mortar mixer and stir for 3 min, then add the cooled activator and continue stirring for 5 min until the mortar is completely uniform. This was poured into the mold and vibrated on the vibration platform for 20 s. After curing in the mold for 24 h, the experimental data were collected after the specimen was cured in saturated Ca(OH)_2_ solution for 28 d.

The test mixing ratio is shown in Table 3. In this orthogonal test, alkali equivalent (D), total volume of RRA (V) and RRA size (S) were used as test variables. To ensure the consistency of aggregate volume in the test, RRA formed equal volume substitution for sand when mixing mortar. D sets three levels, V sets three levels, and S sets four levels. There were 36 test groups in the orthogonal test. In addition, three control groups (EC1, EC2 and EC3) which only contain natural sand and are controlled by D factor were set up. The peak energy consumption of each group measured by SHPB test was set as Ep.

### 2.2. Experimental Program and Analysis

**Figure 1:**
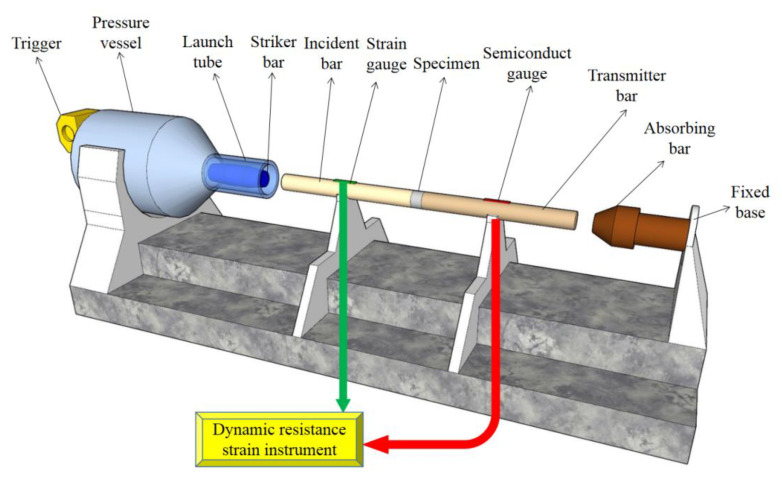
Separating Hopkinson pressure bar system.

The schematic diagram of the SHPB system is shown in Figure 1. A φ50 mm steel rod was used as the material for each impact rod in the experiment. The stress pulse signals propagated in the pressure rod were collected by the strain gauges pasted on the incident rod and transmission rod respectively, and converted into electrical signals by the ultra-dynamic strain gauge; then they were converted into discrete signals by the transient waveform, and were stored for analysis and processing after the completion of the test. The dynamic energy absorption capacity and dynamic compressive strength of RASM specimens can be quantitatively analyzed by the SHPB system.

The diameter of the specimen was consistent with the diameter of the steel rod, and the aspect ratio was 1:2. During the sample preparation, the screened, washed and dried RRA, blast furnace slag powder and natural sand aggregate were fully mixed for 5 min, and then the caustic alkali solution cooled to room temperature was added to the stirrer for wet mixing for 3 min. Finally, the mortar was poured into the mold and cured in a relative air humidity of 95% and 23 degrees for 28 days. After the specimen experienced dynamic load impact, the specimen fragments were collected and screened to obtain the mass of fragments of each size. The screen size is shown in Table 4. After coring the specimen, we soaked it in isopropanol to stop hydration, and conducted SEM tests on the removed samples after drying. The casting surface of the complete specimen was polished completely parallel to the bottom surface, polished with 3000 mesh sandpaper, blackened and dried, and barium sulfate powder was used to image the pores in the section. Statistics of the total number of holes and chord length of specimen cross section were analyzed using a pore structure measuring instrument.

## 3. Results and Discussion

### 3.1. SHPB Absorption Energy Dissipation

#### 3.1.1. Relationship between Compressive Strength and Energy Dissipation

**Figure 2:**
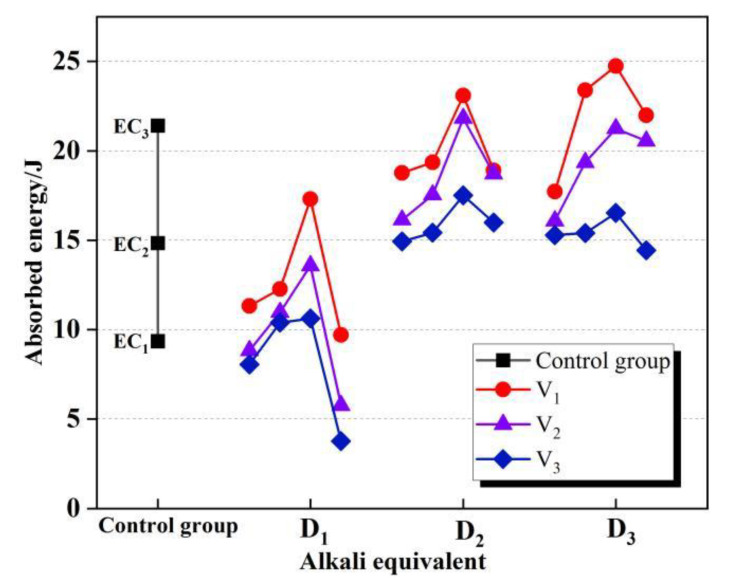
Ep value of RASM.

**Figure 3:**
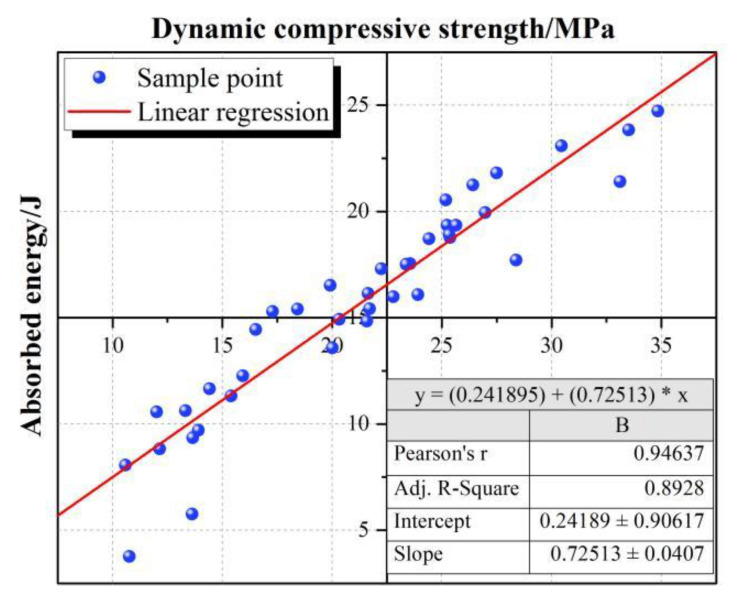
Linear fitting of Ep and dynamic compressive strength of RASM.

**Figure 4:**
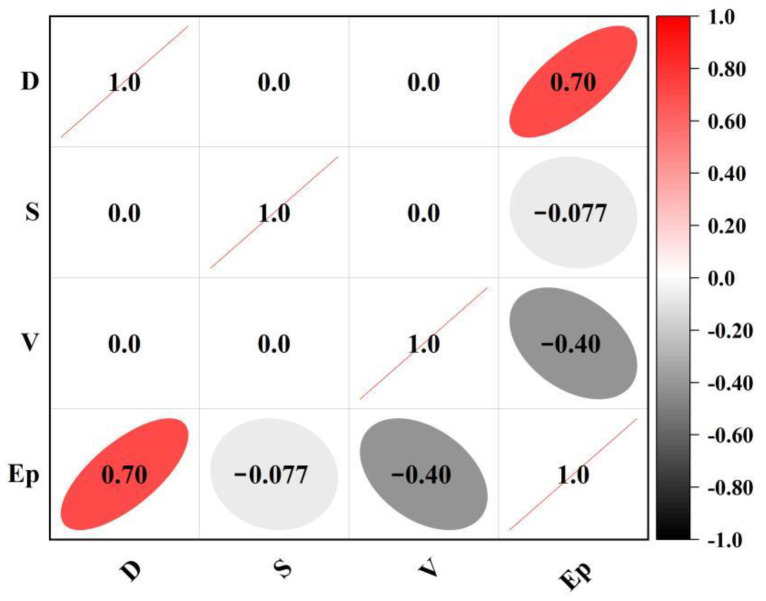
Correlation coefficient matrix.

Dynamic impact experiments were carried out on all experimental groups and three control groups, and the data collected from the experiments were calculated and sorted as shown in Figure 2. The peak compressive strength of each group and the peak dissipated energy were grouped and statistically analyzed, and linear fitting was performed. As shown in Figure 3, the dynamic compressive strength showed a consistent rule with dissipated energy [28], and the two had a positive linear correlation. The relationship between experimental results and factors can be more intuitively demonstrated by the significance and correlation tests. The significance analysis of the three test factors D, V, and S on dissipated energy Ep is shown in Table 4. According to the analysis result, the three test factors all have a significant impact on the dynamic dissipated energy of RASM specimens. The results of the correlation analysis are shown in Figure 4. According to the calculation results of the correlation coefficient matrix, the correlation coefficients of D, V, and S in the interaction of test factors are all 0, completely unrelated, which proves that the three test factors are independent main variables of the experiment and do not interfere with each other. In the dynamic energy absorption test of RASM specimen, D factor has a positive correlation with the dynamic dissipated energy, V factor has a negative correlation with the dynamic dissipated energy, and S factor has no linear correlation with the dynamic dissipated energy.

The transverse comparison of the energy absorption peaks of the experimental groups with the same alkali equivalent showed that with the continuous increase of alkali equivalent, the experimental groups with different V levels showed generally similar regularities but slightly different ones. In the group with V3 level, the peak energy absorption of the specimens increased with the increase of the alkali equivalent, but the slope of the rising curve decreased slightly. In the group with V2 level, Ep continued to increase, but when the alkali equivalent level continued to rise to D3, the peak increase of absorbed energy slowed down, and the overall improvement effect decreased. In the V1 group, this trend was further amplified, and the energy of the D2 group’s absorption peak was higher than D1 and D3. There was a trend of increasing first and then decreasing. After statistical analysis, it was found that the effect of the increase of alkali equivalent on the dynamic energy absorption capacity of the specimen is limited. In the case of high alkali equivalent, the growth of the total volume of RRA led to the gradual enhancement of the negative effect of high alkaline environment on Ep.

#### 3.1.2. Effect of Factor Level on Dissipated Energy

**Figure 5:**
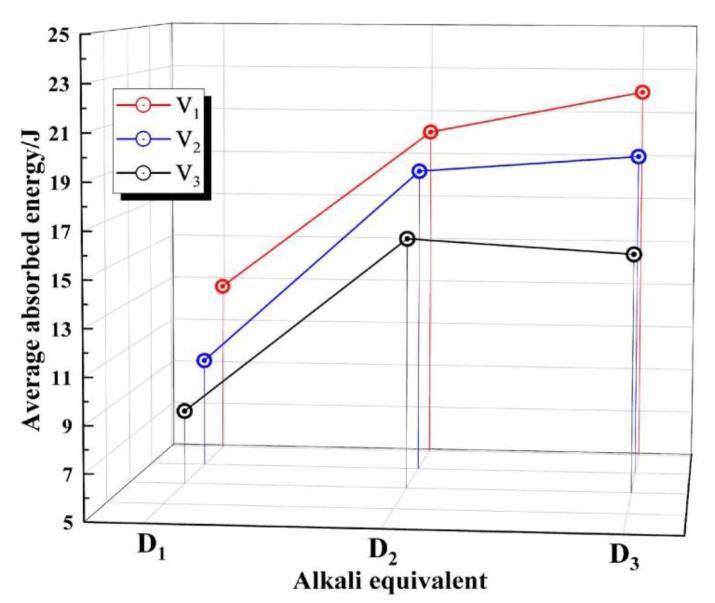
Trend of the average value of Ep.

According to the overall data trends, appropriately increasing the alkali equivalent of RASM specimens will improve the dynamic energy absorption capacity of RASM specimens. This is due to the more complete hydration reaction of alkali excitation blast furnace slag materials. The concrete strength of ascension increases the specimen damage total dissipation, but there is a limit to its promotion. The increase of alkali equivalent increases the amount of C-A-S-H gel produced by RASM, the ability to encapsulate slag particles increases, and the probability of OH^−^ contacting with slag particles decreases. Therefore, the hydration degree of alkali slag cement decreases correspondingly. However, at the same time, with the increase of alkali equivalent, the number of OH^−^ ions also increases, and the vitreous structure of slag is more easily destroyed. The hydration degree of RASM increases first and then decreases with the increase of alkali equivalent. This rule was shown to different degrees in different V factor control groups in this experiment. When the factor level increases from V1 to V3, this trend can be intuitively reflected by the second derivative of the fitted curve, as shown in Figure 5, i.e., the fitted curves are all convex functions, but the rate of slope change of the three curves decreases with the increase of the level of factor V.

**Figure 6:**
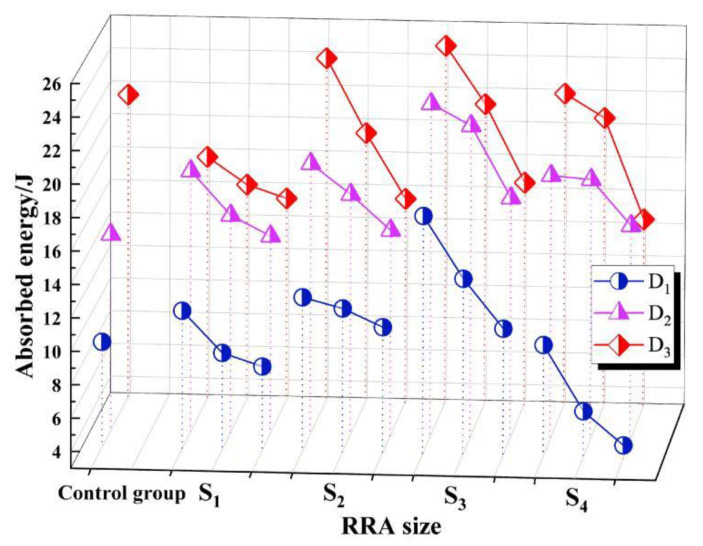
Effect of RRA total volume on Ep.

A horizontal comparison was made between groups with the same amount of RRA, as shown in Figure 6. It can be observed that with the continuous increase of the total amount of RRA, the peak value of dynamic absorbed energy of RASM gradually decreased, which showed a linear rule and did not change significantly due to the influence of other factors. This means that the addition of RRA has a negative effect on the dynamic energy absorption peak of RASM specimens, because RRA itself has no strength, and will not be damaged due to dynamic loading in the experiment. Hence, the increase of the total RRA means the total absorption area of the specimen is reduced, as is the poor RRA as a hydrophilic macromolecule organic matter. Although the high alkaline environment can remove aggregates on the surface of the strong hydrophobic material to strengthen the affinity of the aggregate and cement paste, the strength of the interfacial transition zone of the RRA aggregate is still weaker than that of sand and paste in RASM. The higher the strength of the interfacial transition zone, the higher the energy required for failure. The linear fitting of the data collected from the same group of RRA total amounts shows that the relationship between dissipated energy and factor V was approximately inversely linear in thus experiment.

Differing from the two factors of RRA total amount and alkali equivalent, the influence of the experimental variable RRA size on the test results of the peak dissipation energy does not rise or fall. In Figure 4, when the size of RRA decreases, the dissipation energy of RASM increases first and then decreases, and reaches the peak when the average particle size of RRA is 0.48~0.3 mm. This phenomenon proves that the high alkaline environment has a positive effect on the strength of the interfacial transition zone between RRA aggregate and slurry. When the S factor is at the S1 level, the specific surface area of the same volume aggregate will be lower than other levels, and the specific surface area of RRA increases with the increase of S factor. When the level of the factor reaches S3, the specific surface area is increased compared to S1, and the average Ep at this factor level is 130.8% of the S1 level. However, when the level of S factor continues to increase, that is, the particle size range of RRA is 0.2~0.14 mm, the energy absorption capacity of RASM will be lower than that of S3. There are two reasons for the negative effect. At the S3 level, there are no large bubbles adsorbed in the RASM specimen due to the large aggregate size. In S1, there are a large number of such pores. These harmful holes reduce the uniformity of the specimen and damage the energy absorption capacity of the specimen. Although this phenomenon does not exist in S4, the improvement effect of this factor has reached saturation in S3. On the other hand, the specific surface area of RRA further increases the total ITZ area of RASM specimens. Even if RRA is modified in a highly alkaline environment, its bonding force with the slurry is still low. The weak surface in the specimen increases, so the dynamic energy absorption peak of the specimen decreases in S4.

### 3.2. The Relationship between Absorption Energy and Crushing Morphology

**Figure 7:**
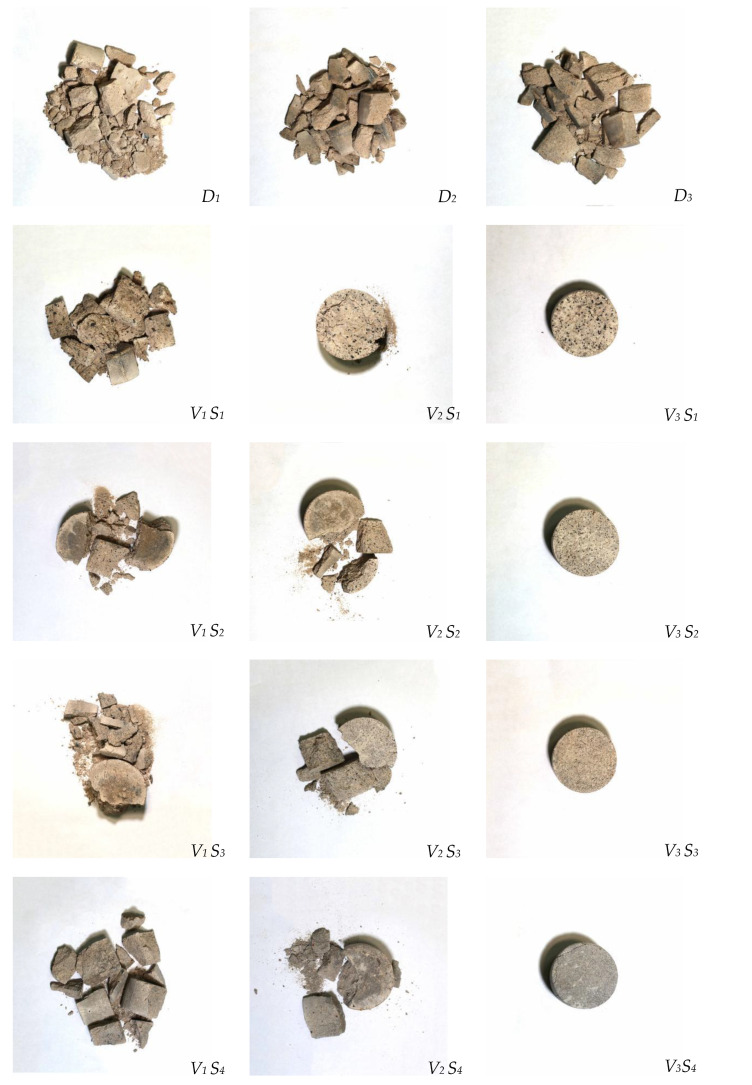
Fracture morphology of RASM specimen.

The fracture morphology of the specimen is shown in Figure 7. RRA is a high-damping viscoelastic high-deformation material [29], and has the dual effect of elastic solid energy storage and viscous liquid energy consumption. In the process of dynamic compression of cement-based materials under impact load, transverse tensile failure or axial compression failure will occur, both of which are brittle failure [26]. The deformation law of RRA in hardened RASM paste is consistent with the change in the macroscopic shape of the specimen. It is a state of transverse tension and axial compression, which will recover after unloading. According to the failure state of the specimen after this test, it is found that the rubber cement mortar specimen will undergo transverse tensile failure when the RRA content is small. The reason for this phenomenon is that in the process of impact compression, due to the transverse unconstrained effect, the specimen changes from the axial stress state to the transverse deformation state to a certain extent. In essence, the specimen itself has no time to release the internal stress through compression failure and presents transverse tensile failure. With the increase of air pressure, the degree of compression failure of the specimen increases and finally gradually transforms into axial compression failure. In the early loading process with more RRA content, the RRA in the specimen is also subjected to tensile and compressive stress and produces tensile and compressive strain, in which the rubber particles absorb energy. In the later unloading process, the rubber particles in the specimen recover to their initial state due to the continuous reduction of tensile and compressive stress. In this process, RRA releases and consumes energy. In the process of impact compression failure of RASM specimens, rubber particles reduce the activated microcracks inside the specimens and hinder the expansion and penetration of microcracks, thus reducing the damage of mortar matrix and reducing the degree of damage. The above analysis is consistent with the fact that the increase of rubber content will increase the average fragmentation, thereby decreasing the fractal dimension calculated by the experimental screening data.

In this experiment, when the level of factor V reaches V2 and V3, the parting dimension of the RASM specimen will not be available because the specimen has been unable to collect separated and uniformly graded fragments. This is shown in Table 5. Although the RRA aggregate reduces the energy absorption capacity of the specimen, at the same time it has the elasticity and recovery ability to reduce the stress concentration of the specimen in the dynamic loading process, which means the RASM specimen with higher RRA content has better crack resistance and seismic capacity, and has a stronger ability to withstand cyclic loading [30].

### 3.3. Microstructure Analysis

#### 3.3.1. SEM Test Procedure

After the RASM specimen underwent dynamic loading, the fragments of the specimen were taken out, immersed in anhydrous ethanol, and the water in the specimen was replaced with anhydrous ethanol to stop the specimen from hydration. This process lasts for 3 days. Subsequently, the treated samples were placed in a vacuum drying oven and taken out after drying for 1 d. After the surface was sprayed with gold, the samples were observed under a scanning electron microscope.

#### 3.3.2. Image Analysis and Discussion

**Figure 8:**
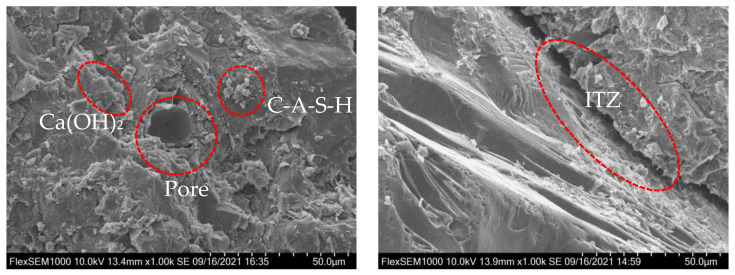
SEM image.

**Figure 9:**
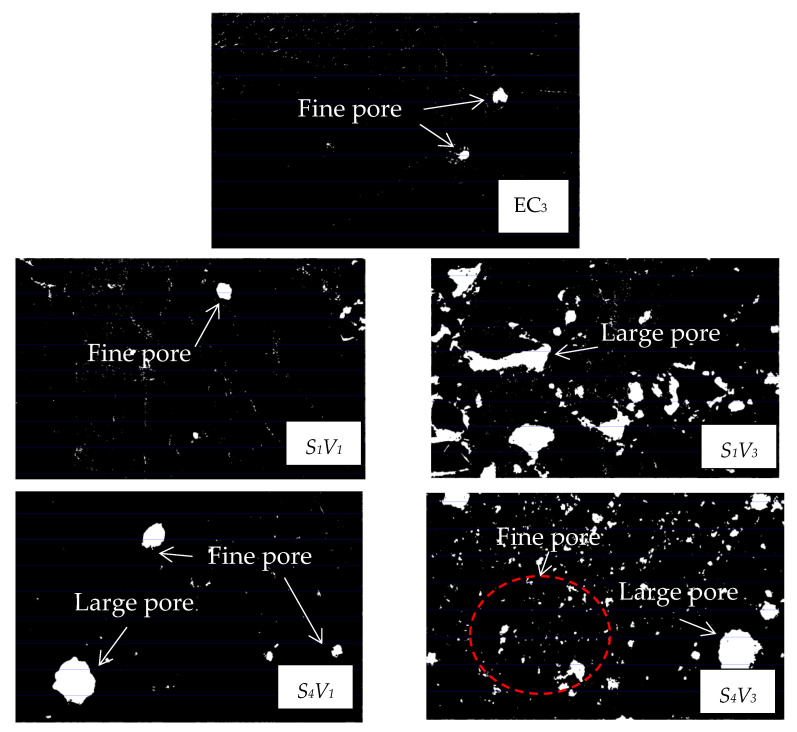
Image of pore morphology.

The RASM specimens were characterized by SEM and the microscopic pore morphology was photographed, as shown in Figure 8 and Figure 9. On the fracture surface of the specimen, it can be observed that RRA is peeled off in a large area, and there is no residual hardened paste on the surface, which proves that the bonding force between RASM and paste is weak, and the specimen tends to crack in the interface transition zone when it is destroyed. The hydration products generated by RASM specimens include C-A-S-H, Ca(OH)_2_ and a small amount of ettringite. When the size of RRA is small, fine pores can be found in the interfacial transition zone between slurry and aggregate at the magnification of 1000× *g*, which increases the porosity of the specimen and reduces the dissipation energy required for failure.

The images collected by the microscopic pore characterization test were binarized. The white area in the picture is the pores in the mortar, while the black part is the hardened slurry. The pore morphology of ASM specimens without RRA is shown in Figure 9. Only natural river sand is used as aggregate in the slurry. There are some small pores in the section of the ASM specimen after condensation, and they all exist independently. By comparing the experimental images, the following rules can be found: the change of D factor level has no obvious effect on the microscopic pore morphology of RASM specimens. When V factor is used as the main evaluation standard, as shown in Figure 9, with the increase of V factor level, the microscopic pore morphology has changed significantly. Comparing the pore characterization of V1 and V4 levels, it can be found that the porosity and gas content of V1 are significantly less than those of V4 at any S factor level. It can be seen that the amount of RRA determines the number of pores carried on the aggregate surface in the RASM specimen. When considering the influence of different levels of S factor on the pore morphology of RASM specimens, it can be found that in the specimens with the same V factor level, there are large coherent pores in the specimens at the S1 level. This is because the RRA in the specimens has a large continuous surface so that large pores can remain on it. At the S2 level, there are still a small number of large pores in the specimens. After the level is raised to S3 and S4, there will be no large pores in the specimens when the total amount of RRA is small. In addition, at the S1 level, the increase of the total amount of RRA will lead to the connection of large independent harmful holes, forming a coherent weak surface, which will have a negative impact on the total amount of energy absorbed by RASM specimens when they are destroyed.

## 4. Conclusions

The dynamic energy absorption capacity of RASM is controlled by three primary factors: the alkali equivalent of cementitious materials, the total amount of RRA and the size of RRA. The dynamic compressive strength measured by the test and the data of the peak absorbed energy showed the same trend. The three main variables of the test were completely orthogonally tested.

The peak dynamic absorption energy of EC3 at D3 level is 128% higher than that of EC1. The experimental group’s highest value of dynamic absorption energy is obtained in D3V1S3. This group is 522% higher than the lowest value group (D1V3S4). These experimental phenomena prove that the main variables in the test have a very significant effect on the Ep value of the RASM specimen.

When the level of D factor increases, the Ep of the specimen generally shows an upward trend, but the difference in the proportion of RRA in the RASM specimen will change this trend. The rate of change in the slope of the Ep mean curve decreases with the increase of the level of V factor. The increase in the level of V factor will lead to a linear decrease in Ep, while Ep under the control of S factor shows the characteristics of increasing first and then decreasing, and reaches the highest value at S3.

RASM undergoes transverse tensile failure or axial compression failure during dynamic compression. RRA exhibits a state of transverse tension and axial compression when it is loaded, and will recover after unloading. When the content of RRA is small, the brittle failure of transverse tension will mainly occur in RASM. When the content of RRA is large, the energy consumed by recovery deformation hinders the expansion and penetration of microcracks.

In the SEM and mesopore characterization tests, RASM contains more harmful pores, which is not conducive to the energy absorption of the specimen during crushing. When the S factor level rises, the size of the pores will decrease. The change of D factor will not change the pore morphology significantly. When the V factor level rises, the pore volume of the specimen increases, and the large-scale RRA leads to the increase of continuous penetrating pores, which further damages the energy absorption capacity of the specimen.

## Figures and Tables

**Table 1:** Main oxide components of RRA.

Oxide Component	SO_3_	SiO_2_	ZnO	Na_2_O	CaO	Al_2_O_3_	Fe_2_O_3_	SrO	Other Oxides
**(%)**	37.62	24.51	15.36	7.02	5.21	3.58	1.83	1.28	3.59

**Table 2:** Main oxide components of S95 slag.

Oxide Component	CaO	SiO_2_	Al_2_O_3_	MgO	SO_3_	TiO_2_	Fe_2_O_3_	K_2_O
**(%)**	36.21	26.24	16.16	13.21	4.82	2.21	0.79	0.24

**Table 3:** Definition and classification of test factors.

Experimental Factors	Level	Definition
Alkali equivalent	D1	2.17 (EC1)
	D2	3.39 (EC2)
	D3	4.7 (EC3)
Total volume of RRA	V1	6%
	V2	12%
	V3	24%
RRA **size**	S1	2.1~1.6 mm
	S2	1.25~0.8 mm
	S3	0.48~0.3 mm
	S4	0.2~0.14 mm

**Table 4:** Significance analysis of main factors.

Source	Sum of Squares	Degree of Freedom	Mean Square	F-Value	Significance
Corrected Model	795.088	7	113.584	41.965	√
Intercept	8923.114	1	8923.114	3296.741	√
D	537.768	2	268.884	99.342	√
S	114.563	3	38.188	14.109	√
V	142.756	2	71.378	26.371	√
Error	75.786	28	2.707		
Total	9793.987	36			

**Table 5:** The fractal dimension of V1 test group.

Group	Fractal Dimension	Group	Fractal Dimension	Group	Fractal Dimension
D1V1S1	2.248	D2V1S1	1.841	D3V1S1	1.605
D1V1S2	2.121	D2V1S2	1.256	D3V1S2	1.106
D1V1S3	1.555	D2V1S3	1.253	D3V1S3	1.074
D1V1S4	1.58	D2V1S4	1.493	D3V1S4	1.34

## Data Availability

The data presented in this study are available on request from the corresponding author.
